# Report of the Bombay Medical Board, Dated July 2, 1802

**Published:** 1803-06-01

**Authors:** W. Moir, H. Scott


### REPORT *of the* BOMBAY MEDICAL BOARD, *dated July* 2, 1802

### *To the Editor of the Bombay Courier.*

FOR the satisfaction of the public, and the information of professional men in India, we beg of you to publish the following account of the introduction of the Cow-pox into this place. We have it now in our power to communicate the benefit of this important Discovery to every part of India, perhaps to China, and the whole eastern world. We shall spare no pains in accomplishing a purpose so desirable, by which one of the greatest evils that has afflicted humanity may be diminished in a great degree, or even extinguished altogether.

In the course of the last twelve months we have repeatedly received by sea, from England, the Vaccine matter, with which many children have been inoculated to no purpose. We were not more successful in matter which was sent to us directly by land from Constantinople. Fortunately, Doctor Short, a surgeon of this Establishment, residing at Bagdad, produced the disease at that place. He immediately forwarded the matter to Bussora, where Mr. Milne, surgeon of that residence, also succeeded in infecting a patient with it. Mr. Milne soon afterwards inoculated a number of other children, and he sent the Vaccine matter to Bombay by several ships. Even with this matter, we were for a time unsuccessful, and after thirty or forty trials by various methods and by different surgeons.

A fortunate inoculation at length produced the Vaccine disease in Anna Dusthill, who is, perhaps, the first human being being who underwent it in India. This child, the daughter of a servant of Captain Hardie, is about three years of age. She is very healthy, and certainly never had the Small-pox. It is necessary to mention these circumstances, as from her alone, the whole of the matter that is about to be sent all over India, was at first derived. We have received no history of the patients from whom it was taken at Bagdad and Bussora, but we rest with confidence, from our knowledge of the medical gentlemen at these places, that no pains have been spared to pass it through unexceptionable bodies.

From Anna Dusthill, on the 8th day of her disease, and on the 22d of last month, seven children were inoculated; five of those, who certainly never had the Small-pox, took the infection, and have already gone through nearly the whole course of the Vaccine disease. The other two were not infected, but there is some probability that one of them has had the Small-pox. From the five children that were infected, about thirty more have been inoculated, and a great many of them, no doubt, will take the disease. From these last we shall send the Vaccine matter to the other presidencies, to Surat, Poona, &c. and care shall be taken that none shall be employed but from an unexceptionable source.

The Vaccine disease in Anna Dusthill, passed, as we have said, through its ordinary course, as described by writers on the subject. The pustule began to show itself about the third day; during the course of the fifth and sixth she had slight symptoms of fever, and some uneasiness in the arm-pit of the inoculated side. The pustule on the eighth day, was of the proper size for that period; it was flat, rather concave, and it consisted of many cells, which on being pricked, gave out a transparent fluid; by the tenth day, the inflamed areola round the pustule was extensive and very distinct in spite of the blackness of her skin. She had only a single pustule on the inoculated part, nor during the whole time did she suffer any material inconveniency from the complaint.

All the five children who were inoculated from her, had a similar train of symptoms; on two of them, whose parents were Europeans, the inflamed areola, from the whiteness of their skin, was much more distinct than it had been on Anna Dusthill. We have thus detailed the progress of the symptoms, and we have, no doubt, but that this is the genuine Cow-pox; some surgeons here, who have seen the disease in Europe, are of the same opinion.

nion. We hope, therefore, that this will tend to quiet the apprehension of parents, which in some instances we find to be very great, and that our experience at this place, so far as it has gone, will give confidence to practitioners. Almost all the medical men at this presidency have witnessed this disease, many of them are inoculating for it, nor do we understand that any difference of opinion has arisen concerning its nature.

One test, indeed, we still want of its genuine nature, and that is, its power of preventing the variolous infection; to this test it shall shortly be put. As this island does not contain less than 150,000 people, sufficient supplies of children must arise to keep up the disease, even without any dependence on Salsette or the neighbouring Continent.

The Hindoos and Parsees, both here and at Surat, show the utmost desire of having their children inoculated with the Vaccine disease. We shall instruct the native practitioners of physic regarding it; but on this part of the subject we are not without apprehensions. Whoever is sufficiently acquainted with what has been done in Europe, with regard to the Cow-pox, is aware that some foreign poison, such as that of the Small-pox, is apt to be mixed with it, when a compound disease arises; or some other poisonous matter may be, from various causes, introduced instead of the Vaccine virus, when a disease altogether different is produced. The History of the Count de Moffet, as lately detailed by Doctor de Carro, affords a most instructive lesson on this subject. The greatest care therefore should be employed to warn the native practitioners, that the Vaccine matter may be degraded by many causes, and that their utmost attention is necessary to prevent it. We can affirm, from our own knowledge, that this government has anxiously assisted our wishes for procuring the Vaccine disease by the way of Bussora. They represented to Lord Elgin, the importance of it to this great society of mankind, and they called for the aid of the Residents of Bussora and Bagdad. Doctor de Carro, of Vienna, who has distinguished himself so honourably in this career, transmitted in the first instance the Vaccine matter to Lord Elgin, who several times before had shown us his attention to the subject; by his Lordship's orders it was sent to our resident at Bagdad, and again to the resident at Bussora. To both these gentlemen the public are under great obligation for the interest they took in the subject. Finally, it fell into the hands of Doctor Short and and Mr. Milne, as we have already said, nor could it have been more fortunately placed.

We have been more particular than was necessary for the medical profession, who must be supposed to be in possession of every fact that has occurred on the subject in Europe; but as we shall disperse the Vaccine disease very widely, as it will affect, and as we hope it will promote the happiness of every family, we wish to satisfy the public at large, concerning the source from which we have derived it, and the foundation of our belief that it is of a genuine kind.

*July 2, 1802.*

W. MOIR.
H. SCOTT.

